# Molecular Characterization of High Mobility Group Box 1a (*HMGB1a*) Gene in Red-Bellied Pacu, *Piaractus brachypomus*

**DOI:** 10.1155/2023/2774528

**Published:** 2023-06-06

**Authors:** Nicolas Carrillo-Godoy, Iang Schroniltgen Rondón-Barragán

**Affiliations:** Laboratory of Immunology and Molecular Biology, Faculty of Veterinary Medicine and Zootechnics, Universidad del Tolima, Santa Helena Highs, Ibagué-Tolima 730006299, Colombia

## Abstract

High mobility group box 1 (HMGB1) is a chromosomal protein in the nucleus and a potent extracellular proinflammatory cytokine, widely described in mammals, nevertheless, with scarce reports in fish. In this study, full open reading frame of *HMGB1a* gene from *Piaractus brachypomus* is reported as well as its molecular characterization, including tissue gene expression. At predicted protein level, *HMGB1a* showed similarities with its orthologs in teleosts and higher vertebrates. The relative gene expression of *HMGB1a* mRNA was measured in several tissues including the brain, where a differential expression appeared in brain regions, i.e., higher expression in the cerebellum and telencephalon. In addition, in an assay of sublethal exposure to chlorpyrifos, upregulation of *HMGB1a* was detected in optic chiasm. Furthermore, in a traumatic brain injury model, upregulation of *HMGB1a* expression was evident 24 hours after lesion and remained higher up to 14 days. These findings suggest a role for *HMGB1a* in brain damage and its candidature as biomarker of brain injury; however, more studies are required to elucidate the functions of *HMGB1a* and its regulation in *P. brachypomus*.

## 1. Introduction

High mobility group (HMG) proteins are a class of non-histone proteins, normally found in the nucleus, that play a role in the regulation of DNA-dependent processes [1]. HMGs are classified into three families: HMGA (HMGAT-hook family), HMGN (HMG-nucleosome-binding family), and HMGB (HMG-box family) [2], the latter being the one with the highest expression [3]. High mobility group box 1 (HMGB1) is highly conserved among vertebrates and can be found in the nucleus, cytoplasm, or in the extracellular milieu [1, 4]. This protein acts as a chromosomal protein in the nucleus, and in the cytoplasm prevents mitochondrial abnormalities, and promotes autophagy, and from cytoplasm, it gets released into the extracellular medium as a danger-associated molecular pattern (DAMP) or secreted by immunocompetent cells in response to different stimuli, such as the recognition by pathogen-associated molecular patterns (PAMPs) or DAMPs. HMGB1 outside the cell, regulates the immune response by stimulating the release of cytokines and promoting the differentiation, proliferation, and maturation of immunocompetent cells [5–8], through interaction with Toll-like receptors (TLR) (TLR2, TLR4, and TLR9) and the endogenous receptor for advanced glycation end-products (RAGE) [4–11]. In addition, HMGB1 is involved in neurogenesis during early development of mammals and fish [8, 9] and neuroinflammation [10], and may contribute to neuropathology and inhibition of neurogenesis through secondary tissue damage.

Recently, HMGB1 has been reported in different aquatic species with similar functions to its mammalian orthologs [3], including its upregulation under pathological conditions [7]. In teleost fish and lampreys, HMGB1 recombinants can interact with DNA and participate in cytokine-like immune responses in the extracellular medium [1, 4, 6, 11]. Furthermore, Fang et al. [12] in a zebrafish model of spinal injury showed that upregulation of HMGB1 promotes neurogenesis, angiogenesis, and recovery of motor capacity and cell survival, and then its downregulation minimizes the effects of inflammation during recovery, which suggest a dual role of this protein in neuroinflammation.

The red-bellied pacu (*Piaractus brachypomus*) is an endemic freshwater fish of economic importance in Colombia, which has been used as a biomodel in pharmacological and immunotoxicological studies [13–16]; nevertheless, more studies are required to validate biomarkers associated with pathologies and disease mechanisms in these native fish species. To the knowledge of the authors, there are no molecular characterization studies of the *HMGB1a* gene and its gene expression in *P. brachypomus*. Thus, the present study aims for the molecular characterization of *HMGB1a* and its transcript expression brain tissue of *P. brachypomus*, after exposure to chlorpyrifos and brain injury.

## 2. Materials and Methods

### 2.1. Ethical Approval

All the experimental procedures followed the guidelines of the Bioethics Committee of the Central Research Directorate of the University of Tolima in the framework of the project code 310130517, based on Law 84/1989 and Resolution 8430/1993, in addition to complying with the parameters established for the care of animals and its use in research and teaching [17, 18].

### 2.2. RNA Extraction and cDNA Synthesis

Total RNA from brain samples for all experiments was extracted using TRIzol reagent (Invitrogen, USA), following the manufacturer's instructions. RNA quality and concentration were assessed by molecular spectrophotometry using NanoDrop™ One (microvolume UV-Vis spectrophotometer, Thermo Fisher Scientific, USA). Finally, cDNA was synthesized using the High Capacity cDNA Reverse Transcription Kit (Thermo Fisher Scientific, USA) following the manufacturer's recommendations. The quality of the cDNA was evaluated by amplifying elongation factor 1 alpha (*EF-1α*) gene by RT-PCR [13].

### 2.3. *HMGB1a* cDNA Sequencing and Primer Design

*HMGB1a* gene sequence was obtained by nanosequencing, using the MinION sequencer (cDNA sequencing kit, SQK-DCS109, Flow Cell R9.4.1, N-50 of 800, Oxford Nanopore Technologies, United Kingdom) from *P. brachypomu*s brain cDNA, mapped on *HMGB1a* sequence from *Colossoma macropomum* (accession number XM_036561022) as a reference template using the Geneious Prime v2023.04 software [19]. Based on the mapped sequence, the primers were designed (Table 1) to amplify the full open reading frame (ORF).

### 2.4. Endpoint PCR Assay

For the PCR reaction, a ProFlex PCR System thermocycler (Applied Biosystems, MA, USA) and GoTaq® Flexi DNA polymerase were used (Madison, USA), following the manufacturer's guidelines. The amplification products were revealed by horizontal electrophoresis using a subcell electrophoresis chamber (Bio-Rad, CA, USA) on a 2% agarose gel stained with HydraGreen™ (ACTGene, NJ, USA) and a molecular weight marker of 100 bp (New England Biolabs, USA). The gel was visualized and documented using the ENDURO GDS Gel Documentation System (Labnet International, NJ, USA). The amplification products were purified and sequenced by the Sanger method (Macrogen Inc., Korea).

### 2.5. Analysis of Sequences and Construction of Model Structures

The sequence of the *HMGB1a* full ORF was confirmed by Sanger sequencing, and amino acid (aa) sequence was predicted using the Geneious Prime software v2023.04 [19], and Expasy ProtParam tool was used for calculation of molecular weight and isoelectric point (pI), among other intrinsic parameters [20]. In addition, the online servers Signa1P 6.0 [21], NetGPI-1.1 [22], DeepLoc-2.0 [23], NetNGlyc [24] and NetOGlyc [25] (DTU Health Tech, Denmark), were used for analysis of putative signal peptide, GPI anchor, most likely location and N-glycosylation and O-glycosylation sites, respectively. Protein domains were predicted using the Conserved Domains tool (NCBI, USA). The *HMGB1a* structural model was constructed in SWISS-MODEL [26], using as template the Cryo-EM structure of mouse RAG1/2 HFC complex containing a partial HMGB1 linker; the model was modified in PyMOL 2.0 [27], to improve its interpretation.

### 2.6. Sequence Alignment and Phylogenetic Analysis

Multiple sequence alignment (MSA) was performed in Geneious Prime software v2023.04 [19], using *HMGB1a* amino acid sequences reported in the GenBank, to *Colossoma macropomum* (XP_306416916), *Pygocentrus nattereri* (XP_017572966), *Silurus meridionalis* (XP_046717257), *Ictalurus punctatus* (XP_017344145), *Tachysurus fulvidraco* (XP_026995128), *Megalobrama amblycephala* (XP_048035832), *Pimephales promelas* (XP_039532079), *Labeo rohita* (ARX80203), *Alosa alosa* (XP_048084920), *Alosa sapidissima* (XP_041949179), *Clupea harengus* (XP_012683898), *Thunnus albacares* (XP_044227023), *Seriola dumerili* (XP_022594514), *Lates calcarifer* (XP_018547270), *Morone saxatilis* (XP_035522445), *Homo sapiens* (NP_001300821), *Macaca mulatta* (NP_001252735), *Mus musculus* (NP_001300823), *Rattus norvegicus* (NP_037095), *Gallus gallus* (XP_040509225), *Anas platyrhynchos* (XP_027325676), *Pseudonaja textilis* (XP_026564649), and *Thamnophis elegans* (XP_032075701). *HMGB1a* phylogenetic tree was built using the neighbor-joining method model of the Geneious Primer v2023.04 software [19] and the Jukes–Cantor genetic distance model and considering 1000 bootstrap hits. In addition, the *Drosophila melanogaster* elongation factor 1 alpha sequence (accession number NP_524611) was set as an outgroup.

### 2.7. Experimental Design

For the treatments in this study, 24 *P. brachypomus* juveniles (2.5 ± 0.3 g) from the same spawning and regardless of the sex, which came from a hatchery, were used. The fish were kept in a 90 L glass tank, with a thermostatically controlled temperature of 25°C, aeration without a filter, and light periods of 12 hours, and fed twice daily with commercial food (30% protein, Solla®) in an amount equivalent to 2% of their body weight. The fish were acclimatized in a period of 15 days and were treated with NaCl to eliminate ectoparasites [28]. Animals were randomly distributed for the experiments, and brain samples were collected.

#### 2.7.1. Sublethal Chlorpyrifos (CPF) Exposure Assay

The animals were divided into two experimental groups: a treatment group (*n* = 5) exposed to a sublethal concentration of CPF (0.011 *μ*g/L) [29] for 72 hours and a control group (*n* = 3) without CPF exposure. To maintain CPF concentrations in the water, half of the water volume in each glass tank was replaced every 24 hours and the corresponding CPF amount was added.

#### 2.7.2. Brain Injury (BI) Assay

Brain injury was performed following the stab wound model [30, 31]. For this, the animals were divided into 4 groups: Group 1 (control group, *n* = 4, 0 h) without puncture, Group 2 (*n* = 4) 24 hours post-injury; Group 3 (*n* = 4) at 7 days post-injury, and Group 4 (*n* = 4) 14 days post-injury. Before brain puncture, the fish were anesthetized by immersion in a glass tank containing 50 mg/L of dissolved eugenol (eugenol, clove oil, Proquident S.A., Colombia) [13] until reaching the state of general anesthesia. The lesion was performed with a 000-caliber sterile entomological needle, with a puncture depth of 5 mm on the left side of the skull until reaching the telencephalon and the optic bulb. The control group was treated with the same manipulation and anesthesia procedure, without puncture, and sacrificed after sedation.

### 2.8. Brain Region Basal Differential Expression

Basal relative expression of the *HMGB1a* in brain regions was measured from brain samples of healthy *P. brachypomus* belonging to a tissue bank collection from our laboratory, where dissected olfactory bulb, telencephalon, optic bulb, hypothalamus, cerebellum, medulla oblongata, and optic chiasm were used. RNA extraction and cDNA synthesis were performed as mentioned above.

### 2.9. Sampling

From each experiment before sampling, fish were anesthetized by immersion in a glass tank with dissolved anesthetic until they reached the stage of general anesthesia [32]. Then, individuals were euthanized by cervical dislocation [33], and brain samples were collected, and the olfactory bulb, optic chiasm, and telencephalon were dissected and collected separately. All tissue samples were snap frozen in liquid nitrogen and stored at −20°C until analysis.

### 2.10. Assessment of *HMGB1a* Transcript Relative Expression by qPCR

For qPCR, primers were designed based on the confirmed ORF sequence of *HMGB1a* from red-bellied pacu (Table 1). qPCR assays were run by duplicate on a QuantStudio 3 real-time PCR system (Thermo Fisher Scientific, USA) following the fast ramp program and using Luna® Universal qPCR Master Mix (New England Biolabs, USA). Each amplicon was validated by melting curve analysis and gel electrophoresis imaging to ensure that there was no amplification of genomic DNA or possible misannealing. Relative gene expression was calculated using the 2^−ΔΔCt^ method [34], and *EF1α* mRNA level was used for normalization.

### 2.11. Statistical Analysis

Data were analyzed by descriptive statistics, and normality was determined by the Shapiro–Wilk test. Differences in gene expression between treatments with CPF were assessed using the Mann–Whitney *U* test, while for the injury treatment and expression in the brain, Kruskal–Wallis test and the two-step augmentation method of Benjamini, Krieger, and Yekutieli as post hoc were used, using GraphPad Prism v 9.0 for MacOS (La Jolla, CA, USA).

## 3. Results

### 3.1. Tissue Expression of *HMGB1a* by RT-PCR Assay

**Figure 1:**
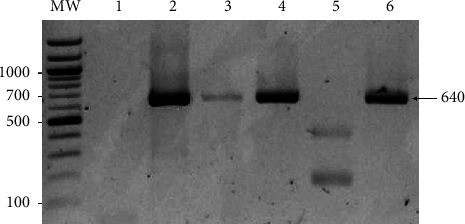
Detection of *HMGB1a* gene (640 bp) in *Piaractus brachypomus* tissues. 1: blood, 2: brain, 3: liver, 4: gill, 5: spleen, and 6: head kidney. MW: 100 bp molecular weight marker (New England Biolabs, USA). Electrophoresis gel made with 2% agarose.

cDNA showed optimal values of quality by biomolecule spectrophotometry, and the reference gene *EF-1α* was detected in all tissues (data not shown). Full ORF of *HMGB1a* from *Piaractus brachypomus* (*PbHMGB1a*) was amplified from the brain, liver, gill, and head kidney, showing a band of the expected size (Figure 1).

### 3.2. Analysis of Sequences and Construction of Model Structures

ORF from *PbHMGB1a* gene corresponds to 612 nt, which codes for a 203 aa protein, with a predicted molecular weight of 23454.18 Da and theoretical pI of 5.96. Mature protein lacks signal peptide and GPI anchor, and it has nuclear location. *PbHMGB1a* showed two domains, a N-terminal HMG-box A domain (Lys_7_-Lys_75_), a central HMG-box B domain (Asn_92_-Arg_162_) and acidic C-terminal tail with abundant glutamic and aspartic acids residues (Asp_181_-Glu_203_). Within the sequence are two cysteines (Cys_22_, Cys_44_) in the HMG-box A domain and one cysteine (Cys_105_) in the HMG-box B domain. Two N-glycosylation sites (Asn_36_-Phe_37_-Ser_38_ and Asn_133_-Lys_134_-Thr_135_) and two O-glycosylation sites (Ser_14_ and Thr_175_) were predicted. Nucleotide sequence was submitted to GenBank (NCBI, USA) under accession number OQ330857.

**Figure 2:**
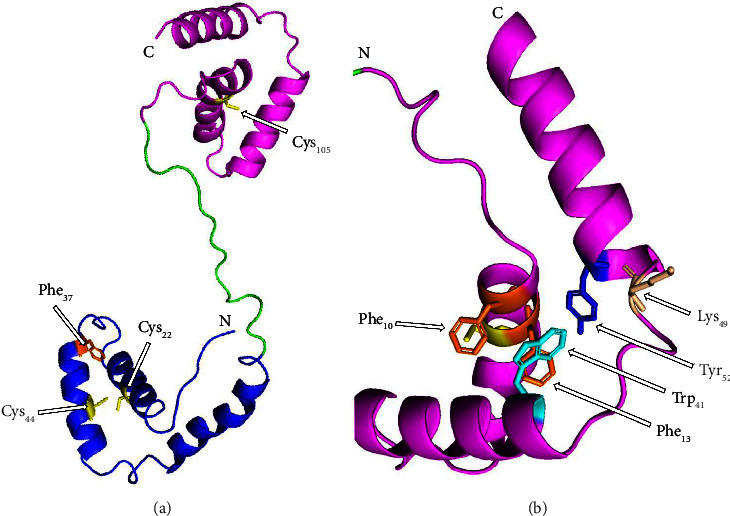
Predicted cartoon model of the *PbHMGB1a* protein. The structure of the protein was predicted using the BIOZENTRUM SWISS-MODEL tool. (a) The domains of the protein and the presence of *α*-helices and cysteines (Cys) and phenylalanine (Phe) are illustrated. (b) HMG-box B domain and location of phenylalanine (Phe), tryptophan (Trp), lysine (Lys), and tyrosine (Tyr). HMG-box A domain (blue), HMG-box B (pink), the cysteines (yellow), the phenyl rings (orange), tryptophan (light blue), lysine (white), tyrosine (blue), N-terminus (N), and C-terminus (C).

A model of the protein was predicted using the Cryo-EM structure of mouse RAG1/2 HFC complex containing partial HMGB1 linker as a template, with an identity of 88.89% and a Global Model Quality Estimate of 0.53 (Figure 2). The secondary structure of the protein reveals the presence of 7 *α*-helices, of which 4 are in the HMG-box A domain and 3 in the HMG-box B domain.

### 3.3. Sequence Alignment and Phylogenetic Analysis

**Figure 3:**
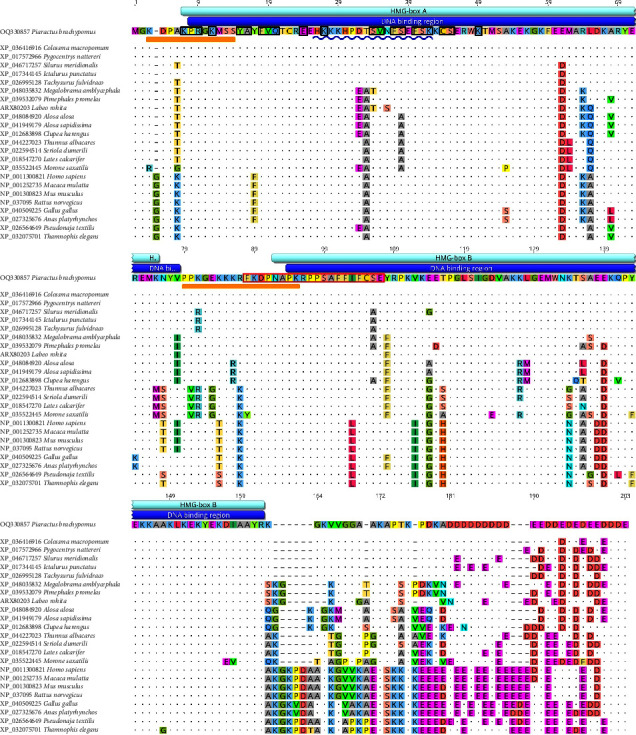
Multiple alignment of the amino acid sequences of *PbHMGB1a* among teleost fish and higher vertebrates. Domains are highly conserved. Protein domains (light blue), DNA-binding regions (dark blue), nuclear localization signal (green), LPS-binding regions (yellow line), p53-binding regions (black boxes), and TLR4-binding region (red box).

The MSA of the sequences showed an identity between *PbHMGB1a* and 16 orthologous sequences from different orders of fish in a range from 74.27 to 98.52%, with the higher identity with *Colossoma macropomum*. On the other hand, PbHMGB1a showed a range from 73.61 to 77.03% identity with its higher vertebrate orthologs (Figure 3). In addition, the MSA demonstrates few variations in the sequences of the domains, as well as in the C-terminal acid tail, the latter presenting more residues in higher vertebrates (Figure 3).

**Figure 4:**
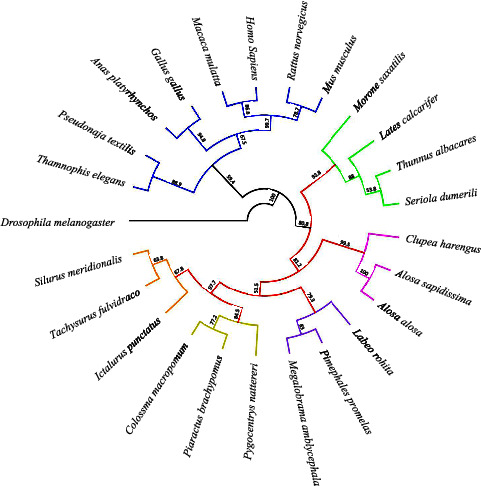
Phylogenetic analysis of HMGB1a sequences from fish and higher vertebrates. The phylogenetic tree was built using the neighbor-joining method and 1000 bootstrap. Fish sequences are grouped separated from mammals. Within the teleost clade, a grouping of different orders is observed, each represented by a different color (green = perciformes; pink = cupleiformes; violet = cypriniformes; yellow = characiformes; orange = siluriformes), thus *PbHMGB1a* is directly related to the characiform species, *Piaractus brachypomus* (OQ330857) *Colossoma macropomum* and *Pygocentrus nattereri*. The numbers at the nodes indicate the bootstrap values. Sequences of *HMGB1a* correspond to *Colossoma macropomum* (XP_306416916), *Pygocentrus nattereri* (XP_017572966) *Silurus meridionalis* (XP_046717257), *Ictalurus punctatus* (XP_017344145), *Tachysurus fulvidraco* (XP_026995128), *Megalobrama amblycephala* (XP_048035832), *Pimephales promelas* (XP_039532079), *Labeo rohita* (ARX80203), *Alosa alosa* (XP_048084920), *Alosa sapidissima* (XP_041949179), *Clupea harengus* (XP_012683898), *Thunnus albacares* (XP_044227023), *Seriola dumerili* (XP_022594514), *Lates calcarifer* (XP_018547270), *Morone saxatilis* (XP_035522445), *Homo sapiens* (NP_001300821), *Macaca mulatta* (NP_001252735), *Mus musculus* (NP_001300823), *Rattus norvegicus* (NP_037095), *Gallus gallus* (XP_040509225), *Anas platyrhynchos* (XP_027325676), *Pseudonaja textilis* (XP_026564649), *Thamnophis elegans* (XP_032075701).

From the phylogenetic analysis, two clearly defined clades were obtained, in which the *HMGB1a* sequences of fish are grouped, separated from those of higher vertebrates (Figure 4). On the other hand, subgroups of fish belonging to the same taxonomic order are formed, which demonstrates the evolutionary difference of the protein between the different orders of teleost fish.

### 3.4. *PbHMGB1a* mRNA Expression

**Figure 5:**
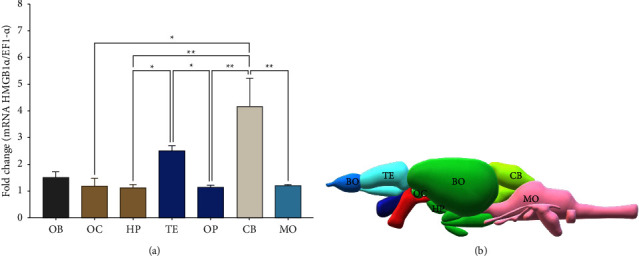
Relative expression of *PbHMGB1a* in different regions of the brain. (a) OB = olfactory bulb; OC = optic chiasm; HP = hypothalamus, TE = telencephalon; OP = optic bulb; CB = cerebellum; MO = medulla oblongata. *EF-1α* was used as a reference gene. ^*∗*^*p* < 0.05 and ^*∗∗*^*p* < 0.01. Data are expressed as average ± SEM. (b) Graphical representation of anatomical regions of the brain of *P. brachypomus* analyzed for *HMGB1a* expression.

The levels of *PbHMBG1a* mRNA were measured in brain regions of *P. brachypomus*, showing a higher expression in the telencephalon and cerebellum (Figure 5(a)). The expression in the telencephalon and cerebellum was higher compared to the hypothalamus, optic bulb, and, and medulla oblongata.

**Figure 6:**
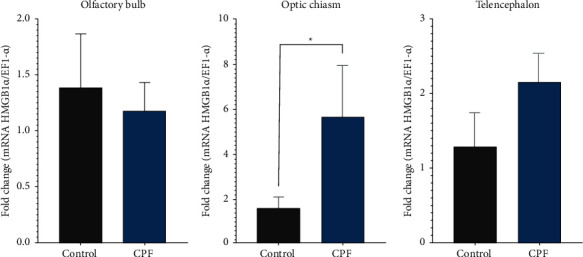
Relative expression of *PbHMGB1a* transcripts after 72 hours of exposure to CPF. *EF-1α* was used as a reference gene. Data are expressed as average ± SEM. ^*∗*^*p* < 0.05.

**Figure 7:**
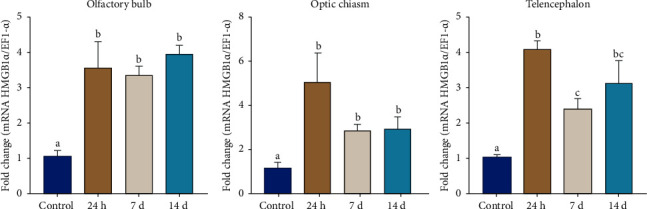
Relative expression of *PbHMGB1a* after brain injury. *EF-1α* was used as the reference gene. Data are expressed as average ± SEM. Different letters denote differences statistically significant between groups (*p* < 0.05).

On the other hand, *PbHMGB1a* mRNA level was higher in individuals exposed to CPF compared to the control group in the optic chiasm (*p* < 0.05) (Figure 6). In the case of brain injury, *PbHMGB1a* mRNA was upregulated at 24 hours and 7 and 14 dpi compared to the control group (*p* < 0.05) in the three tissues evaluated. However, in the telencephalon at 7 days post-injury, *PbHMGB1a* mRNA levels decreased compared to levels observed at 24 hours and then increased slightly at 14 days post-injury (Figure 7).

## 4. Discussion

The high mobility group box (HMGB) family of proteins was originally discovered in the bovine thymus [5] and is divided into four subgroups, HMGB1, HMGB2, HMGB3, which share high identity, and HMGB4 lacking in identity acidic C-terminus [3, 7, 35]. HMGB1 was identified as a ubiquitous bifunctional protein [1], which in the nucleus acts as a non-histone chromatin-associated factor [11], binds to DNA in a structure-specific manner, stabilizes nucleosome formation, aids in error repair, and plays important roles in gene transcription in both higher vertebrates and teleosts [1, 6, 7]. Pathogenic stimulation and oxidative stress induce the nucleocytoplasmic translocation of HMGB1 [4], which in grass carp induces heat shock protein 70 (HSP70) to move to the nucleus where it interacts with HMGB1b generating the nucleocytoplasmic translocation of the protein and activation of the HMGB1b-Beclin 1-mediated autophagy [4]. However, outside the cell, HMGB1 is a linker of innate and acquired immunity, as it acts as a cytokine [1, 5], through interaction with pattern recognition receptors such as TLR and RAGE [4, 11], leading to activation of the primary myeloid differentiation response 88 protein (MyD88) dependent on the NF-kB pathway [36]. In addition, it plays a role in apoptosis, cell migration, and cytoskeleton reorganization [1].

In teleosts, HMGB presents a similar activity, demonstrating the capacity to stimulate the respiratory burst, the production of nitric oxide, and the proliferation of immunocompetent cells, as well as increasing the release of proinflammatory cytokines (TNF-a and IL-1b), by mediating the activity transcription of NF-kB [7, 11]. On the other hand, HMGB1 expression increases after exposure to pathogens and prevents replication of the pathogen [6, 7]. They suggest that HMGB1 activates the immune response after injury or infection by stimulating the release of cytokines and the activation of immune cells. The early increase in *PbHMGB1a* expression 24 hours after brain injury indicates that it may be released due to cell injury and could be involved in the initiation of inflammation; however, whether *PbHMGB1a* can modulate the immune response remains to be clarified like their teleost counterparts.

### 4.1. Sequence Analysis

HMGB1 is bipolar [7], has two positively charged folded helical DNA-binding domains (HMG-box A and B), and a negatively charged C-terminal acid tail rich in glutamic and aspartic acids [11], which regulates the interaction between domains, as well as other nuclear proteins with DNA [7, 35]. *PbHMGB1a* presents these domains which are highly conserved in vertebrates; the length, molecular weight, and isoelectric point are similar to those previously described in other teleosts and lampreys [1, 5–7, 11]. On the other hand, the C-terminus of higher vertebrates has acidic residues that are not present in any of the teleosts, including *P. brachypomus*; however, it is believed that the length of this region may not be critical for the biological function of HMGB1 [7].

Box A has been shown to contain receptor-binding sites [37], its reduced form is anti-inflammatory, and binding to the C-terminus of the protein increases this activity [35]; however, it presents two cysteines (Cys23, Cys45), which mediate autophagy by interacting with Beclin 1 and form a disulfide bond under mild oxidative conditions [38], which gave it inflammatory activities [5]. Box B is a proinflammatory domain [37] and presents a cysteine (Cys106) that promotes cytoplasmic localization and is related to TLR4 activation and TNF-*α* release [1]. Likewise, these components are involved in the nucleocytoplasmic translocation of HMGB [4]. The presence of cysteines (Cys22, Cys44, and Cys105) in *PbHMGB1a* supports the possible role of this protein in immunity, as has been described in other teleosts and lampreys [1, 5, 39], due to the presence of these cysteines in positions corresponding to those described in mammals.

The absence of a signal peptide in *PbHMGB1a* indicates that it may be secreted via an alternative pathway that bypasses the endoplasmic reticulum and the Golgi apparatus [7] via acetylation of lysine residues [2]; however, the presence of HMGB1 in plasma membranes has been reported, which is associated with a non-classical secretory signal peptide, the 18 N-terminal amino acids of hydrophilic acylated surface protein B (HASPB) [35].

Two N-glycosylation (Asn_36_-Phe_37_-Ser_38_ and Asn_133_-Lys_134_-Thr_135_) and O-glycosylation (Ser_14_ and Thr_175_) sites were predicted in PbHMGB1a. Glycosylation alters proteolytic resistance, protein solubility, stability, local structure, circulating lifetime, and immunogenicity; specifically, N-glycosylation and O-glycosylation modify secreted and membrane proteins [24, 25]. On the other hand, post-translational modifications such as acetylation, phosphorylation, methylation, ADP-ribosylation, and oxidation have been shown to be involved in nucleocytoplasmic translocation [35]. These findings suggest that the interaction between different post-transcriptional modifications ultimately determines the location of HMGB1, which remains to be demonstrated for glycosylation.

The predicted PbHMGB1a structure revealed 7 *α*-helices distributed in the domains, like those of *Scophthalmus maximus* L., whose secondary structure was composed of 9 *α*-helices, 7 *β*-turns, 2 *γ*-turns, and other structures [3]. In humans and other mammals, the presence of *α*-helices generates two loops in both domains, conferring an L-shape, presenting a higher content of *α*-helices in HMG-box A [35], like what was observed in the present model. The aromatic amino acids Phe_10_, Phe_13_, Trp_41_, Lys_49_, and Tyr_52_ in HMG-box B and their distribution within the secondary structure of PbHMGB1a (Figure 2(b)) demonstrate that they could interact to give the L-shape to this domain, since they are found in same positions to those reported for humans, where Phe_14_, Phe_17_, Trp_45_, Lys_53_, and Tyr_56_ are found at the junction between the two helical arms [40].

On the other hand, the HMG-box A domain presents structural changes in its oxidized form, related to disulfide bonds in Cys_23_ and Cys_45_ that displace the N-terminus of helix II, and this allows hydrophobic interaction between the phenyl rings of Phe_38_ [38]; given the presence of these amino acids in similar positions (Figure 2), it is likely that these interactions occur in *PbHMGB1a*.

These similarities in sequence and structure suggest that *PbHMGB1a* may exhibit behavior and function like those described in humans and other mammals [35]. This is supported by the evidence of few residue variations in the domain/binding sites compared with human HMGB1 (P09429), except for the RAGE-binding site which showedlower identity, and some amino acids are absent in *PbHMGB1a*.

### 4.2. Phylogenetic Analysis

MSA reveals high percentages of identity between vertebrate and teleost HMGB sequences, which could be the result of low selective pressure for evolutionary maintenance of amino acid sequences [14]. Analysis of the constructed phylogenetic tree indicates that *PbHMGB1a* is highly related to its teleost orthologs, grouping in a different clade from higher vertebrates, which is consistent with other phylogenetic analyses of teleost HMGB [3, 5]. The phylogenetic relationships also demonstrate the evolutionary divergence between the different orders of teleosts, where each one of these is grouped in a different clade, and thus PbHMGB1 is grouped with the species of Characiformes, *Colossoma macropomum* and *Pygocentrus nattereri*.

### 4.3. Gene Expression

HMGB1 is ubiquitously distributed in teleosts, being found in the kidneys, gills, brain, blood, heart, liver, spleen, intestine, muscle, and skin [5–7]; however, the site of highest expression varies according to the species [3, 5–7, 11]. This wide distribution, under normal conditions, suggests a fundamental role of HMGB1 in teleosts [7]. In this study, the absence of *HMGB1a* in the blood and spleen samples of *P. brachypomus* may indicate lower expression in the tissue below the limit of detection of conventional PCR.

On the other hand, differential expression of PbHMGB1a in brain regions evidenced highest expression in the cerebellum and the telencephalon. This can be explained by neurogenesis in the teleosts which occurs in the telencephalon and cerebellum [8, 9, 41], where the HMGB1 plays a pivotal role. In contrast with mammals, the expression of HMGB1 is almost null in the brain of adult mammals and the neurogenesisis limited to certain regions [35],such as the granular cells of the gyrus dentatus of the hippocampus, in the olfactory bulb, in certain areas of the telencephalic ventricles, and in almost all nuclei of the granular cells of the cerebellar cortex [8, 9]. HMGB1 expression in the mammal cerebellum is relevant in granular cells, since it can interact with RAGE and the human natural killer cell glycan (HNK-1) to stimulate migration and growth of neurites in these developing animal cells, regulating downwards after the migration [8]. In teleosts, granular cells are the main population of constantly proliferation in the cerebellum [41], which may explain the high expression of PbHMGB1a in this region, which is related to the migration of granular cells, as well as in the remodeling of neuronal circuits in the red-bellied pacu.

HMGB1 may play an important role in the mammalian and teleost nervous system as it is involved in early brain development, cell migration, and neurite formation [2, 8]. Similarly, it is important in neurogenesis and angiogenesis after injury; however, its release produces multiple inflammatory and neurotoxic factors, contributing to secondary tissue damage from injury [12].

In our study, *PbHMGB1a* mRNA levels increase in the telencephalon 24 hours after the lesion and then decrease at 7 dpi, maintaining similar levels to 14 dpi. However, *PbHMGB1a* mRNA levels in the optic chiasm and olfactory bulb remain high during the experiment, possibly due to factors related to the site of the lesion and its severity. In zebrafish, *Danio rerio*, HMGB1 mRNA increases shortly after a lesion in the central nervous system, associated with the stimulation of neurogenesis and angiogenesis; however, it undergoes a rapid decrease, preventing the effects of inflammation, resulting in increased cell survival and less tissue damage during recovery [12], which is similar to the results in the present study. Likewise, in an extra-neuronal tissue, the valve intestine of the Siberian sturgeon, *Acipenser baerii* and the challenge with *Streptococcus iniae*, induced an upregulation of HMGB1 expression in the acute phase followed by a downregulation [5].

On the other hand, CPF, an organophosphate, is stable in water and can be rapidly absorbed through the gills, skin, and intestinal tract, causing neurotoxicity, hepatotoxicity, immunotoxicity, oxidative stress, and alterations in blood biochemical parameters [42], along with effects on neurobehavioral and locomotor behavior [43, 44]. In *P. brachypomus*, subchronic exposure to CPF increased the density of astrocytes, modified their morphology, and increased the expression of biomarkers related to reactivity [45]. In *Clarias gariepinus*, multifocal vacuolization was generated in the brain after exposure to CPF [46]. In the present study, the *PbHMGB1a* mRNA levels increased in the optic chiasm after sublethal exposure to CPF, possibly due to its high blood supply [15]; it could have been exposed to higher concentrations of the pesticide, generating greater injury and expression of *PbHMGB1a*. The effects of CPF could also be increased given the tank temperature (25°C) despite the low dose and short exposure time, since exacerbation of the effect has been reported at high temperatures [44].

Because fish can be exposed to different concentrations of pesticides in aquatic ecosystems close to places of agricultural activity [44], the study of molecular biomarkers of neuronal and tissue damage after exposure to pollutants may help to assess the environmental impact of the contamination and establish proper and accurate measurements.

## 5. Conclusion

Full ORF of *PbHMGB1a* was detected which allowed its bioinformatic and phylogenetic analysis. Expression analysis showed a wide distribution of the gene in red-bellied pacu tissues as well as its response in toxicity and brain injury models, which denote it as a candidate brain biomarker in the central nervous system, and due to its differential expression distribution in brain regions, further studies are needed to describe its role in pathophysiology of specific brain areas.

## Figures and Tables

**Table 1:** Sequences of the primers used for the amplification of *HMGB1a* and *EF-1α* from *Piaractus brachypomus*.

Gene	Sequence (5′-3′)		Tm (°C)	Amplicon size (bp)
*HMGB1a* (ORF)	F	GGGACATCTTCAACATGGGG	58.23	640
	R	CCAATTTTTACTCATCATCGTCCTC	58.28	
*HMGB1a* (qPCR)	F	CTGCAGAGAGGAACACAAGAA	57.88	198
	R	CTCTTCTTCTTCTCGCCTTTG	56.65	

*EF-1α*	F	ACTGAGGTCAAGTCTGTGGA	57.91	110
	R	CCACGACGGATGTCTTTAA	54.96	

*HMGB1a*, high mobility group box 1a; qPCR, quantitative polymerase chain reaction; *EL-1α*, elongation factor 1 alpha.

## Data Availability

The data used to support the findings of this study are available from the corresponding author upon request.
